# Associations of Migration, Socioeconomic Position and Social Relations With Depressive Symptoms – Analyses of the German National Cohort Baseline Data

**DOI:** 10.3389/ijph.2023.1606097

**Published:** 2023-07-18

**Authors:** Nico Vonneilich, Heiko Becher, Barbara Bohn, Berit Brandes, Stefanie Castell, Andreas Deckert, Nico Dragano, Claus-Werner Franzke, Amand Führer, Sylvia Gastell, Halina Greiser, Thomas Keil, Carolina Klett-Tammen, Lena Koch-Gallenkamp, Lilian Krist, Michael Leitzmann, Claudia Meinke-Franze, Rafael Mikolajczyk, Ilais Moreno Velasquez, Nadia Obi, Annette Peters, Tobias Pischon, Marvin Reuter, Tamara Schikowski, Börge Schmidt, Matthias Schulze, Dmitry Sergeev, Andreas Stang, Henry Völzke, Christian Wiessner, Hajo Zeeb, Daniel Lüdecke, Olaf von dem Knesebeck

**Affiliations:** ^1^ Institute of Medical Sociology, Center for Psychosocial Medicine, University Medical Center Hamburg-Eppendorf, Hamburg, Germany; ^2^ Heidelberg Institute of Global Health, Heidelberg University Hospital, Heidelberg, Baden-Württemberg, Germany; ^3^ NAKO e.V., Heidelberg University, Heidelberg, Baden-Württemberg, Germany; ^4^ Leibniz Institute for Prevention Research and Epidemiology (LG), Bremen, Germany; ^5^ Department of Epidemiology, Helmholtz Center for Infection Research, Helmholtz Association of German Research Centers (HZ), Braunschweig, Niedersachsen, Germany; ^6^ Institute for Medical Sociology, University Hospital of Düsseldorf, Düsseldorf, Germany; ^7^ Institute for Prevention and Cancer Epidemiology, University of Freiburg Medical Center, Freiburg, Baden-Württemberg, Germany; ^8^ Institute for Medical Epidemiology, Biometrics and Informatics (IMEBI), University Hospital in Halle, Halle, Saxony-Anhalt, Germany; ^9^ NAKO Study Center, German Institute of Human Nutrition Potsdam-Rehbruecke (DIfE), Potsdam, Brandenburg, Germany; ^10^ Division of Cancer Epidemiology, German Cancer Research Center (DKFZ), Heidelberg, Baden-Württemberg, Germany; ^11^ Institute of Social Medicine, Epidemiology and Health Economics, Charité University Medicine Berlin, Berlin, Germany; ^12^ Institute of Clinical Epidemiology and Biometry, Faculty of Medicine, University of Würzburg, Würzburg, Bavaria, Germany; ^13^ State Institute of Health I, Bavarian Health and Food Safety Authority, Erlangen, Germany; ^14^ Department of Clinical Epidemiology and Aging Research, German Cancer Research Center (DKFZ), Heidelberg, Baden-Württemberg, Germany; ^15^ Deptartment of Epidemiology and Preventive Medicine, University Medical Center Regensburg, Regensburg, Bavaria, Germany; ^16^ Institute for Community Medicine, University Medical Center Greifswald, Greifswald, Mecklenburg-Vorpommern, Germany; ^17^ Molecular Epidemiology Research Group, Max Delbrück Center for Molecular Medicine, Helmholtz Association of German Research Centers (HZ), Berlin, Baden-Wurttemberg, Germany; ^18^ Institute for Occupational and Maritime Medicine Hamburg, University Medical Center Hamburg-Eppendorf, Hamburg, Hamburg, Germany; ^19^ Institute of Epidemiology, Helmholtz Center München, Helmholtz Association of German Research Centres (HZ), Neuherberg, Germany; ^20^ Berlin Institute of Health (BIH), Charité University Medicine Berlin, Berlin, Germany; ^21^ Subject Sociology, University of Bamberg, Bamberg, Bavaria, Germany; ^22^ Leibniz-Institut für Umweltmedizinische Forschung (IUF), Dusseldorf, Germany; ^23^ Institute for Medical Informatics, Biometry and Epidemiology, Essen University Hospital, Essen, North Rhine-Westphalia, Germany; ^24^ German Institute of Human Nutrition Potsdam-Rehbruecke (DIfE), Potsdam, Brandenburg, Germany; ^25^ Institute of Nutrition Science, Faculty of Mathematics and Natural Sciences, University of Potsdam, Potsdam, Brandenburg, Germany; ^26^ Institute of Medical Biometry and Epidemiology, University Medical Center Hamburg-Eppendorf, Hamburg, Hamburg, Germany

**Keywords:** German National Cohort, NAKO, migrant health, migration, social relations, depressive symptoms, socioeconomic position, social integration

## Abstract

**Objectives:** We analyze whether the prevalence of depressive symptoms differs among various migrant and non-migrant populations in Germany and to what extent these differences can be attributed to socioeconomic position (SEP) and social relations.

**Methods:** The German National Cohort health study (NAKO) is a prospective multicenter cohort study (N = 204,878). Migration background (assessed based on citizenship and country of birth of both participant and parents) was used as independent variable, age, sex, Social Network Index, the availability of emotional support, SEP (relative income position and educational status) and employment status were introduced as covariates and depressive symptoms (PHQ-9) as dependent variable in logistic regression models.

**Results:** Increased odds ratios of depressive symptoms were found in all migrant subgroups compared to non-migrants and varied regarding regions of origins. Elevated odds ratios decreased when SEP and social relations were included. Attenuations varied across migrant subgroups.

**Conclusion:** The gap in depressive symptoms can partly be attributed to SEP and social relations, with variations between migrant subgroups. The integration paradox is likely to contribute to the explanation of the results. Future studies need to consider heterogeneity among migrant subgroups whenever possible.

## Introduction

The prevalence of depressive symptoms and adverse mental health is affected by recognized risk factors, including female gender, advanced age, lower socioeconomic status, and weak social connections [1, 2]. However, these factors may not act on their own but often in combination [3]. There is compelling evidence for a socioeconomic gradient in depression. It has been shown that lower levels of education, income or occupational status are generally associated with higher risks of depression and adverse mental health [4–6]. This association was found in different age groups [7–9]. There is also evidence for poorer mental health of populations in countries with higher income inequality [7–9] and for socioeconomic inequalities in treatment outcomes of depression [10].

Furthermore, social relations have been linked to both mental health and depressive symptoms. In their systematic review, Santini et al. identified different aspects of social relations to be associated with depressive symptoms. Highest risks for depressive symptoms were found for those who perceived their social support as inadequate [11]. Being socially isolated has also been identified as risk factor for depressive symptoms and adverse mental health [12].

Persons with migration background often carry higher mental health risks [13–15]. Possible reasons include socioeconomic disadvantage, harmful working and living conditions, experience of racism and discrimination and traumatic experiences in countries of origin, during migration or after arrival in host-countries [3, 16]. Various studies have shown that migrants and their descendants often report lower social integration and a lack of social support [14, 16, 17]. Social integration and social support enhance subjective wellbeing and can serve as a buffer against acute stress and adverse mental health effects [18–20]. Earlier studies found that social relations contribute to reduced mental health risks of migrants [11, 14, 21, 22]. In a Swedish study, Brydsten et al. [14] identified lack of social activity and low social support, among others, as key factors explaining higher mental health risks of migrants compared to non-migrants. Lecerof et al. [22] found that trust in others and social participation had a protective effect on mental health when migrant populations under study faced financial difficulties or experienced discrimination. The heterogeneity within migrants has often been neglected or not been analyzed, mostly due to reasons of data availability and the lack of including migrants in research.

People with migration background are not a homogenous group but instead consist of a broad range of nationalities, cultures, origins, languages, experiences and socioeconomic determinants [23, 24]. The concept and definition of migration background in Germany has been described in detail by Will [25]. Various historical, social and political peculiarities need to be considered in the history of migration in Germany. A general comparison of migrant versus non-migrant populations neglects these differences [23, 24, 26]. Most of the studies mentioned above regarding mental health gaps and the social disadvantage of migrant populations have rarely addressed the heterogeneity of persons with migration background [26–28]. In 2019, 21.2 million people in Germany (about 26% of the total population) can be classified as 1^st^ generation migrants with own migration experience (about 13.7 million people) or as descendants of first-generation migrants who were born in Germany, so called 2nd generation migrants (about 7.6 million people; [29]). The largest groups are 1st generation migrant workers who came to Germany under political agreements between 1950 and the 1970s, especially from Turkey and countries in Southern Europe (Italy, Spain, Portugal, and Greece). Together with their descendants, they account for about 6.8 million people. Another frequent region of origin of migrants in Germany is Eastern Europe, especially from countries such as Poland and Romania. Among those with origins in Eastern Europe and the former Soviet Union are the so-called German resettlers, who automatically received German citizenship upon federal recognition as resettlers. About 4.6 million resettlers migrated to Germany since 1950 [29].

The aim of this study is to consider this heterogeneity in migrants and their descendants and to go into more detail regarding findings on mental health and their possible explanations among different groups of people with migration background in Germany. Our analyzes are based on data from the German National Cohort (NAKO), which, due to its size of more than 200,000 study participants, allows a differentiated analysis of the health situation of migrant populations in Germany [28].

Based on the aforementioned background, the following research questions are analyzed: (I) Are there differences in depressive symptoms between non-migrant and different migrant populations? (II) Can these migration-related differences in depressive symptoms be attributed to social integration, emotional support and socio-economic position (SEP)?

## Methods

The NAKO is a prospective multicenter cohort study funded by the Federal Ministry of Education and Research, the federal states and the Helmholtz Association [30]. Main goal of the NAKO is to investigate risk factors and causes of common chronic diseases. Data collection and assessment is organized by 18 study centers across Germany, covering rural and urban areas with both high and low population density [28, 31, 32]. The NAKO included 204,878 participants aged 20–69 at baseline, based on random samples from data of the population registration authorities of the respective study centers, stratified by sex (1:1) and age (10.0% each 10-year group between 20 and 39 years, and 26.7% in each 10-year group between 40 and 69 years). The overall response at baseline was 17%, but varied between 9% and 32% across the 18 different study centers [33]. Baseline assessments took place from March 2014 until September 2019. These included face-to-face interviews, self-administered questionnaires, various physical examinations and the collection of biospecimens in all study centers, including whole-body 3T magnetic resonance imaging of about 30,000 participants. The wide range of assessments and the average data collection time of up to 5.6 h contributed to the 5 years time span of the baseline examination [33]. Further details on sampling and data assessment can be found in earlier publications [31, 32]. The sample used in the present analyses consists of 204,878 respondents, of which 34,108 can be classified as migrants. All participants provided written consent for study participation and all study centers’ local ethical committees gave their approval. The complete study was carried out in accordance with the Declaration of Helsinki.

### Measures

#### Dependent Variable

Depressive symptoms were assessed using the Patient Health Questionnaire PHQ-9 [34]. The scale is well established and has been used in many other studies, including former research using the NAKO data [35, 36]. The PHQ-9 consists of nine items that relate to depressive symptoms during the past 2 weeks. Each item has a four-point-scale, ranging from 0 (“not at all”) to 3 (“almost every day”) resulting in a sum score with a range from 0 to 27. Cronbach’s Alpha for the PHQ-9 scale was 0.84, showing a high internal consistency of the scale. A validated cut-off score of 10 points or higher indicates depressive symptoms in terms of a moderate to severe depressive episode [37]. We used the dichotomized PHQ-9 variable as indicator for high (PHQ-9-score ≥10) or low (PHQ-9-score <10) depressive symptoms.

#### Independent Variables

Age and sex of respondents were recorded, where age was divided into three age groups (39 years and younger, 40–59 years, 60 years and older), as depressive symptoms are likely to vary in different age groups and to make results comparable across different risk factors included in the models [38]. Education and income were considered as indicators for socio-economic position (SEP). Education was assessed based on the ISCED-97 classification and recoded into three levels (“low” = ISCED-level 1/2, “intermediate” = ISCED-level 3/4 and “high” = ISCED-level 5/6) [39]. Equivalent household income was calculated based on information of the size of household and income, originally assessed with 16 income categories. Based on this information, relative income position was calculated on a 5-point scale and ranged from 1 (“below 60% of average equivalent income”) to 5 (“above 150% of average equivalent income”). This classification was based on the net equivalent household income from the European Union Statistics on Income and Living Conditions (EU-SILC). For this study, income position was recoded into three categories (“below 60% of average equivalent income,” indicating risk of poverty as defined by EU-SILC; “60%–150% of average equivalent income”; and “above 150% of average equivalent income,” indicating high income groups). As unemployment has been associated with effects both on social relations (e.g., in the work context) and income, we added employment status to our models. This variable indicated whether a respondent was employed, unemployed or non-employed. Pensioners fall into the latter category while self-employed persons fall into the employed status category.

Quantitative and qualitative dimensions of social relations were represented by the social network index measures (SNI, [40]) and emotional support. The SNI contains information on partnership (living with a partner), number of close social contacts (close friends and relatives) and membership in voluntary organizations. The SNI sums this information in a levelled score from 1 to 4, with lowest level 1 indicating social isolation [40]. The four-level SNI was dichotomized for the analyses (score of 1 coded as 1 and all others coded 0), with 1 indicating “social isolation” and 0 “not being isolated”. Emotional support was assessed by asking “Can you count on someone to support you emotionally (e.g., talk to you about problems, help you make a decision)?”. Based on the response categories, it was possible to distinguish to what extent help was needed at all and, if so, to what extent it was perceived as sufficient or insufficient. This variable was also dichotomized: The answers “no” and “yes, but would have needed more support” were recoded into “insufficient emotional support,” while “yes, but had no need” and “yes, and support was sufficient” were recoded into “sufficient emotional support”.

Migration was assessed according to a set of basic indicators for mapping migrant status in Germany [41]. We followed the proposal by Wiessner et al. for the NAKO data enabling more specific analyses and comparisons with non-migrant populations [28]. Migration status was recorded based on participants’ nationality and country of birth and their parents’ nationality and country of birth. This enabled a distinction between non-migrant populations and so-called 1st - and 2nd generation migrants. Based on this information, migrants were divided into two subgroups of 1st generation migrants, resettlers and one subgroup of 2nd generation migrants: Those who have another than a German citizenship; those who were born in another country than Germany but obtained German citizenship; those who can be defined as German resettlers, and those who were born in Germany and have at least one parent with non-German country of birth (2nd generation migrants) [28]. Information on countries of birth of study participants and on countries of birth of their parents were used to generate a variable on regions of origin, based on the 15 regions defined by the United Nations [42], in order to enable more specific analyses and to identify possible heterogeneity within migrant populations. In 1st generation migrants their own country of birth was used for generating the region of origin variable, in 2nd generation migrants the country of birth of the parents was used. When both parents had different countries of birth from different regions, country of birth of the father was used for generating the region of origin variable. The three most common regions across the four different migrant subgroups and their descendants were selected, while the 12 remaining regions were coded as “other” category (see Table 1).

**TABLE 1:** Sample description according to migration status and largest regions of origin (German National Cohort (NAKO), Germany, 2014–2019).

	N	%
Non-migrants	170,770	83.35
1st generation migrants, other than German citizenship	10,525	5.14
Eastern Europe	2,153	1.05
Southern Europe	1,885	0.92
West Asia	1,601	0.78
Other regions	4,886	2.38
1st generation migrants, German citizenship	10,752	5.24
Eastern Europe	4,977	2.43
Southern Europe	784	0.38
West Asia	1,522	0.74
Other regions	3,469	1.69
2nd generation migrants	9,358	4.57
Eastern Europe	1,290	0.63
Southern Europe	1,444	0.70
West Asia	959	0.47
Other regions	5,665	2.77
Resettlers (Eastern Europe)	3,473	1.70
All migrants, total	34,108	16.6
Total	204,878	100

### Missing Data

On average, about 6.5% of individual items across all variables were missing. The variables sex and age had no missing values, while the highest proportion of missing values were found in the variables emotional support (16.3%) and SNI (16.6%). The missing data pattern was analyzed, and missing data was imputed using the multivariate imputation by chained equations method [43]. The method for imputing missing values depends on the variable’s nature. For continuous variables, predictive mean matching was applied, while logistic regressions were used for binary variables and polytomous logistic regression for categorical variables with more than two levels.

### Statistical Analysis

Sample description is reported in terms of means and standard deviations for continuous data and proportion for categorical variables. Results are shown for each migrant group separately and for the total sample. Two logistic regression models with PHQ-9 (dichotomized) as dependent variable and migration as independent variable were calculated, each with a different set of further covariates. In the first model, we included age and sex as covariates. The second model introduced all variables simultaneously and thus included migration, age, sex, SEP indicators (education, and income positions), employment status and social relations indicators (emotional support, SNI). Analysis of collinearity revealed no problems, neither for education and income nor for other control variables used in our models.

We then calculated two more logistic regression models that included the specific regions of origin for different migrant subgroups, which generally replicated the initial models, in order to investigate differences between them and their association with depressive symptoms. For all models, Nagelkerke’s pseudo-r-squared is reported. All analyses were carried out using the R statistical package [44].

## Results

16.6% (*n* = 34,108) of the NAKO sample can be categorized as migrants or their descendants (see Table 1). Further specification revealed that 5.1% (*n* = 10,525) are 1st generation migrants with other than German citizenship, 5.2% (*n* = 10,752) became German citizens after birth, 4.6% (*n* = 9,358) are 2nd generation migrants (German-born with at least one parent of other than German origin) and 1.7% (*n* = 3,473) are German resettlers. When looking at regions of origin, Eastern Europe (e.g., Poland and Rumania), Southern Europe (e.g., Italy) and West Asia (e.g., Turkey) were the three regions of origin named most often across all different migrant categories.

14.3% of 1st generation migrants with non-German citizenship reported 10 years or less of education, while in the non-migrant population only 1.7% do so (see Table 2). A similar trend was found in relative income position. For example, 37.1% of 1st generation migrants with non-German citizenship were attributed to the lowest income position, thus being at risk of relative poverty, while 12.7% of non-migrants were at risk of relative poverty. The proportion of respondents reporting unemployment was highest in migrants and their descendants, with 14.3% of 1st generation migrants with non-German citizenship reporting unemployment. When looking at the two indicators of social relationships, three out of four migrant groups under study showed higher risks of being socially isolated (with the exception of resettlers) and a higher percentage of inadequate emotional support compared to non-migrants. Lack of emotional support was reported by almost every fifth 1st generation migrants with non-German citizenship, social isolation was reported by 17.7% of 1st generation migrants with non-German citizenship. An increased burden of depressive symptoms was detected in all migrants and their descendants compared to non-migrants in Germany. This was true for mean scores of the PHQ-9 questionnaire as well as a score of 10 or higher, indicating the presence of moderate to severe depressive symptoms.

**TABLE 2:** Descriptive statistics of indicators used in the analysis; baseline data (German National Cohort (NAKO), Germany, 2014–2019).

Variables	Non-migrants (*n* = 170,771)	1st generation migrants, other than German citizenship (*n* = 10,544)	1st generation migrants, German citizenship (*n* = 10,730)	2nd generation migrants (*n* = 9,329)	Resettlers (*n* = 3,504)	Total (*N* = 204,878)
Sex, % female, (95%-CI)	50.4 (50.2, 50.7)	50.3 (49.4, 51.3)	49.9 (48.9, 50.8)	49.4 (48.4, 50.4)	56.4 (54.8, 58.1)	50.5 (50.2, 50.7)
Age until 39 years, %, (95%-CI)	19.7 (19.5, 19.9)	26.0 (25.2, 26.9)	13.4 (12.7, 14.0)	29.4 (28.4, 30.3)	28.8 (27.3, 30.3)	20.3 (20.1, 20.5)
Age 40–59, %, (95%-CI)	52.4 (52.1, 52.6)	56.7 (55.7, 57.6)	57.9 (57.0, 58.9)	49.4 (48.4, 50.5)	51.2 (49.5, 52.9)	52.7 (52.5, 53.0)
Age 60+, %, (95%-CI)	27.9 (27.7, 28.1)	17.3 (16.6, 18.0)	28.7 (27.8, 29.5)	21.2 (20.4, 22.0)	20.0 (18.7, 21.3)	26.9 (26.8, 27.1)
ISCED low education, % 10 years or less, (95%-CI)	1.7 (1.7, 1.8)	14.3 (13.6, 15.0)	7.0 (6.5, 7.5)	2.8 (2.5, 3.2)	5.0 (4.3, 5.7)	2.8 (2.7, 2.8)
ISCED high education, % more than 15 years, (95%-CI)	56.5 (56.2, 56.7)	54.4 (53.4, 55.4)	53.0 (52.1, 54.0)	57.1 (56.1, 58.1)	53.9 (52.3, 55.6)	56.2 (55.9, 56.4)
Employment status, % unemployed, (95%-CI)	2.9 (2.8, 3.0)	9.6 (9.0, 10.1)	5.5 (5.1, 5.9)	3.6 (3.2, 3.9)	6.4 (5.6, 7.2)	3.4 (3.4, 3.5)
Employment status, % non-employed, (95%-CI)	20.1 (19.9, 20.3)	18.6 (17.9, 19.3)	22.3 (21.5, 23.1)	18.7 (17.9, 19.5)	14.8 (13.7, 16.0)	20.0 (19.8, 20.2)
Relative income position, % <60% of equivalent income, (95%-CI)	12.7 (12.6, 12.9)	37.1 (36.2, 38.0)	23.3 (22.5, 24.1)	16.9 (16.2, 17.7)	29.4 (27.9, 30.9)	15.0 (14.8, 15.2)
Relative income position, % >150% of equivalent income, (95%-CI)	26.3 (26.1, 26.5)	14.5 (13.8, 15.1)	17.0 (16.3, 17.7)	25.7 (24.8, 26.6)	9.0 (8.1, 10.0)	24.9 (24.7, 25.1)
Social Network Index, % isolated, (95%-CI)	13.3 (13.1, 13.5)	17.7 (17.0, 18.5)	14.1 (13.5, 14.8)	17.5 (16.7, 18.2)	13.0 (11.8, 14.1)	13.7 (13.6, 13.9)
Emotional support, % no/inadequate support, (95%-CI)	8.4 (8.3, 8.5)	19.0 (18.2, 19.7)	14.7 (14.1, 15.4)	10.7 (10.1, 11.3)	12.0 (11.0, 13.1)	9.5 (9.3, 9.6)
PHQ-9 (cut-off >10), % depressed, (95%-CI)	7.0 (6.8, 7.1)	9.2 (8.6, 9.7)	9.8 (9.2, 10.4)	9.9 (9.3, 10.5)	9.1 (8.2, 10.1)	7.4 (7.3, 7.5)
Mean PHQ-9 (SD)	3.85 (3.58)	4.63 (3.91)	4.48 (3.98)	4.40 (4.00)	4.67 (3.71)	3.96 (3.65)

In terms of research question one, results showed significantly increased risks of reporting depressive symptoms for all migrants compared to non-migrants (see Table 3, Model 1). Highest risks were found for both groups of 1st generation migrants (German citizenship OR 1.57; other than German citizenship OR 1.51). Regarding research question two, we found that after controlling for social relations and SEP, in two out of four migrant groups significantly higher ORs remained compared to non-migrants. In non-German citizens and resettlers, the increased risks of reporting depressive symptoms decreased or reversed while this was not the case for those who obtained German citizenship after birth (OR 1.18) and for descendants of migrants (OR 1.28).

**TABLE 3:** Logistic regression models to examine associations of different migration characteristics with depressive symptoms (dichotomized PHQ-9, 1 = score ≥10 indicating depressive symptoms); N = 204,878 (German National Cohort (NAKO), Germany, 2014–2019).

Parameter	Model 1	Model 2
	Odds Ratio (CI)	Odds Ratio (CI)
(Intercept)	0.05 (0.04, 0.05)	0.02 (0.01, 0.02)
1st generation migrants, other than German citizenship (ref. non-migrants)	1.51 (1.39, 1.64)	0.88 (0.81, 0.96)
1st generation migrants, German citizenship (ref. non-migrants)	1.57 (1.47, 1.67)	1.18 (1.10, 1.27)
2nd generation migrants (ref. non-migrants)	1.42 (1.32, 1.52)	1.28 (1.19, 1.38)
Resettlers (ref. non-migrants)	1.40 (1.24, 1.59)	1.09 (0.95, 1.26)
Age 20–39 years (ref. age 60+)	1.58 (1.50, 1.67)	1.95 (1.83, 2.08)
Age 40–59 (ref. age 60+)	1.49 (1.42, 1.56)	1.94 (1.83, 2.06)
Sex (female, ref. male)	1.51 (1.46, 1.56)	1.51 (1.45, 1.56)
Education (ISCED medium, ref. ISCED high)		1.28 (1.23, 1.33)
Education (ISCED low, ref. ISCED high)		1.36 (1.23, 1.50)
Unemployed (ref. employed)		1.97 (1.81, 2.14)
Non-employed (ref. employed)		1.58 (1.49, 1.67)
Income position (60%–150% of equivalent income, ref. >150% of equivalent income)		1.26 (1.20, 1.33)
Income position (<60% of equivalent income, ref. >150% of equivalent income)		1.80 (1.69, 1.93)
Emotional support (insufficient emotional support, ref. sufficient emotional support)		4.52 (4.32, 4.73)
Social Network Index (isolated, ref. not isolated)		1.88 (1.78, 1.99)
Nagelkerke’s Pseudo r-squared	0.02	0.13

In Table 4, migrants and their descendants were further differentiated by region of origin. Resettlers were not included (see results in Table 3) as their region of origin is mostly Eastern Europe, with little exceptions. Compared to non-migrants, migrants and their descendants with origin in West Asia showed highest odds of reporting depressive symptoms with odds ratios ranging from 2.17 to 2.70. Subpopulations with origin in Eastern Europe and Southern Europe showed comparably less elevated odds. While for those 1st generation migrants without German citizenship the introduction of SEP and social relations led to a reduction and even reversal of risks compared to non-migrants, especially with origin in West Asia, this was not the case for 2nd generation migrants with origins in West Asia. After introducing both SEP and the two indicators of social relations, odds for depressive symptoms in 2nd generation migrants with origin in West Asia remained more than two-fold compared to non-migrant populations (OR 2.15; Table 4, Model 2). Similar results were found for 1st generation migrants with German citizenship with origin West Asia, even though the odds ratio after controlling for both social relations and SEP was comparably lower (OR 1.42).

**TABLE 4:** Logistic regression models to examine associations of different migration characteristics with depressive symptoms (dichotomized PHQ-9, 1 = score ≥10 indicating depressive symptoms), specified by region of origin; *N* = 190,731 (German National Cohort (NAKO), Germany, 2014–2019).

Parameter	Model 1	Model 2
	OR (95% CI)	OR (95% CI)
(Intercept)	0.05 (0.04, 0.05)	0.02 (0.01, 0.02)
1st generation migrants, other than German citizenship^a^		
Eastern Europe	1.29 (1.12, 1.50)	0.81 (0.69, 0.95)
Southern Europe	1.52 (1.27, 1.82)	0.95 (0.78, 1.15)
West Asia	2.17 (1.84, 2.56)	0.77 (0.64, 0.92)
Other regions	1.42 (1.26, 1.59)	0.95 (0.85, 1.06)
1st generation migrants, German citizenship^a^		
Eastern Europe	1.38 (1.25, 1.53)	1.24 (1.12, 1.38)
Southern Europe	1.49 (1.18, 1.88)	1.25 (0.96, 1.62)
West Asia	2.52 (2.19, 2.90)	1.42 (1.20, 1.68)
Other regions	1.46 (1.29, 1.65)	0.98 (0.86, 1.12)
2nd generation migrants^a^		
Eastern Europe	1.34 (1.09, 1.64)	1.21 (0.98, 1.49)
Southern Europe	1.45 (1.22, 1.72)	1.26 (1.05, 1.51)
West Asia	2.70 (2.25, 3.24)	2.15 (1.74, 2.65)
Other regions	1.22 (1.10, 1.36)	1.14 (1.02, 1.27)
Nagelkerke’s Pseudo r-squared	0.02	0.13

^a^ Regression models (see Table 3 as reference); Model 1 adjusted for age and sex; Model 2 adjusted for all covariates.

## Discussion

### Summary and Interpretation

Based on data of the German NAKO, we found higher prevalence of depressive symptoms in all migrant groups under study compared to non-migrant populations. These findings are in line with previous larger population studies [14, 22, 45–47]. For example, Brydsten et al. showed that risks of mental distress was higher among various migrant groups compared to natives, based on the Swedish Health on Equal Terms survey [14]. Similarly, Aichberger et al. found higher risks for depression in various migrant groups from different regions in Europe compared to non-migrants based on the SHARE data [45].

Furthermore, it was investigated to what extent these differences can be attributed to socio-economic indicators and social relations. The confounding effects of SEP and social relations varied in different populations: The higher odds ratios of depressive symptoms in 1st generation migrants without German citizenship disappeared or even reversed when SEP and both aspects of social relations were controlled for. This was not the case in 1st generation migrants with German citizenship and in 2nd generation migrants. A differentiation by region of origin detected further variations in associations of migration background and depressive symptoms. Moreover, variations in the role of SEP and social relations for these associations were identified. Highest odds ratios of reporting depressive symptoms compared to non-migrants were found especially in migrants with origin in West Asia, independent of migrant generation or citizenship and not attributable to SEP or social relations. This is in line with previous findings that revealed higher degrees of somatization and stronger symptom severity in Turkish migrants in Germany [26].

The introduction of social relations and SEP into regression models showed varying effects on the associations of migration and depressive symptoms. A possible explanation for the persistence of higher risks for depressive symptoms in 1st generation migrants with German citizenship and in 2nd generation migrants after the introduction of SEP and social relations is that being born in Germany or having German citizenship may be associated with a stronger sense of belonging and a stronger desire for social acceptance [48]. Their continuous refusal, the feelings of not belonging and a higher likelihood of experiencing discrimination may translate into higher risks of poorer mental health [17, 49, 50]. This has been labelled as a form of social defeat, which is associated with higher health risks due to increased stress levels [51]. Earlier studies detected highest degrees of perceived discrimination in Turkish migrants, especially in second-generation migrants and in women [48, 49]. Perceived discrimination and the awareness of relative deprivation are reasons for the integration paradox which states that this process is even more likely to occur in the higher educated and structurally integrated immigrants [52]. It describes that economically more integrated and often higher educated 2nd generation migrants are more likely to turn away from the host society, psychologically. The perception of not belonging might lead to a stronger orientation towards acculturation styles that can be labelled as separation [48]. In contrast, denying or not acquiring German citizenship may be associated with lower hopes for long-term integration, possibly including greater involvement in migrant communities. Differences in risks for poorer mental health are more likely to be explained primarily by socioeconomic disadvantage and lower social integration in these migrant populations, while in 2nd generation migrants, in 1st generation migrant with German citizenship and to a certain degree in German resettlers the effects of SEP and social integration on the mental health gap are considerably lower due to the above-described integration paradox and its consequences.

A healthy migrant effect might play a role in explaining the lower risks of 1^st^ generation migrants without German citizenship. Selection effects such as in-selection, which leads to more homogenous and often healthier groups of migrants leaving their countries of origin, and out-selection, which might favour those economically better off to stay in host-countries are likely. In-selection only affects those with own migration experience and out-selection is potentially more likely for those without German citizenship, which has been labelled as “unhealthy re-migration effect” [53]. These effects might contribute to the differences observed in the likelihood of depressive symptoms in different migrant populations [54, 55].

When looking at social integration in migrant subgroups, it might be valuable for future studies to differentiate between bonding (within one’s own community) and bridging (with other local communities) social capital. For example, the ability to share common cultural practices for refugees within their own community (binding capital) was positively associated with quality of life and mental health [56]. Nonetheless, active commitment within communities and especially across different communities and successful integration in host societies is severely hampered when relevant resources (language, economic, cultural etc.) are lacking and when other needs regularly outweigh the need to socially meet and connect. These effects potentially vary between different migrant subgroups.

Furthermore, future studies need to take a more nuanced look at the migration aspect. Our results vary depending on which specific population of migrants is considered. The more detailed analyses show differences with regard to mental health risks within different migrant populations, and they also indicate that different aspects are relevant for different migrant populations in terms of reducing the risks of depressive symptoms compared to non-migrant populations. Analyses that fail to consider subgroups within migrant populations would fail to detect these differences [57].

### Strengths and Limitations

These analyses of the NAKO data are limited by various aspects. The four items of emotional support and SNI, included in a computer-assisted personal interview at the end of the NAKO assessment, have a comparably high proportion of missing data [31, 33]. Analyses of missing data showed that older and less educated persons as well as those a with migration background were more likely to not participate in the computer-assisted interview. Thus, data were not missing completely at random. Applying multiple imputation can reduce bias and improve efficiency of statistical results [58, 59]. Moreover, we additionally calculated all regression models with original (per-protocol) data that included missing values. We found no patterns that indicate any introduced bias due to imputation of missing data. The average assessment time per participant of up to 5.6 h leads to high quality data and a rich amount of information, but on the other hand also makes baseline assessment overall very time-consuming. Such a high average assessment time makes an early termination more likely for older-aged persons, persons with impaired health or with lower language skills, as mentioned above. Moreover, baseline data collection took more than 5 years (from March 2014 until September 2019). It cannot be ruled out that changes and events during this time period affected the outcome examined here.

Another limiting aspect is the survey language of the NAKO, which is German, without options for translation or the provision of other foreign languages [28]. This systematic underrepresentation of non-German speaking groups may lead to an underestimation of differences in depressive symptoms, as these groups are more likely to face greater challenges in social and socioeconomic integration (e.g., labour market) due to language barriers (see for example, [60, 61]). Data on language proficiency was collected at the end of the interviews, but was not available for the analyses conducted here.

Furthermore, as discussed above, experiences of discrimination and racism were not assessed in the NAKO study. Both aspects and their subjective perception are important determinants of (mental) health and of (successful) social integration, especially in migrant populations [62, 63]. Brydsten et al. [14] found that experiences of discrimination were an explanatory factor regarding the mental health gap especially for non-European migrants. Additional relevant migration-related indicators have recently been proposed for future research on migration and health in Germany, including reasons for migration, self-reported discrimination, social support and sense of belonging to society [24]. Therefore, the neglect of subjective experiences of discrimination and racism remains a blind spot in the NAKO study. These experiences should be addressed and integrated in future studies.

Conceptual overlap has to be considered when interpreting the association between social relations and depressive symptoms. Social isolation is an aspect that is also included in the PHQ-9 [34]. Thus, in a way, social relations are included in the independent and in the dependent variable. Finally, although the NAKO is designed as a prospective cohort-study, only data from the first wave was available for analysis. This cross-sectional design impedes causal interpretations. Reverse causation cannot be ruled out.

Major strength of the NAKO is the sample size. With over 200,000 participants and a wide range of indicators of health, SEP, social relations and many other it offers large potential for social epidemiology. Especially regarding migrant populations, the NAKO allows a differentiation within the same dataset which is still rare. Regions of origin, citizenship, country of birth and information on parents’ country of birth offer the possibility to specify research questions regarding certain migrant populations and overcome the more general comparison of one migrant and one non-migrant population.

### Conclusion

Based on data from the NAKO study in Germany, higher risks of depressive symptoms were found in migrants and their descendants than in non-migrant groups. After the inclusion of SEP, SNI and emotional support in the regression models, the effect of migration status on depressive symptoms remained visible but was reduced, indicating the important role of SEP and social relations for this mental health gap. Both the risks of depressive symptoms and the effects of SEP and social relations varied considerably, when different migrant subgroups and regions of origin were taken into account. This is an important message for future studies in social epidemiology concerning comparisons of migrant and non-migrant populations, as this heterogeneity within migrants and their descendants should be taken into account, whenever possible. The NAKO data offer the potential of differentiated analysis. In the near future, data of the NAKO follow-up will be available and the cross-sectional analysis of the present study should be replicated in a longitudinal-design.
